# Remote neuromuscular electrical stimulation upregulates MDK to enhance macrophage efferocytosis *via* LRP1 in wound healing

**DOI:** 10.7555/JBR.38.20240375

**Published:** 2026-03-19

**Authors:** Lijuan Zong, Chong Liu, Li Zhang, Xueyou Tao, Qingyan Tian, Xiaokai Zhou, Yu Wang, Na Shen, Jiaming Gong, Qingyuan Zhuang, Tong Wang, Wentao Liu, Ying Shen, Liang Hu

**Affiliations:** 1 Department of Anesthesiology, Children's Hospital of Nanjing Medical University, Nanjing, Jiangsu 210008, China; 2 Department of Pharmacology, School of Basic Medical Sciences, Nanjing Medical University, Nanjing, Jiangsu 211166, China; 3 Department of Anesthesiology, Yangzhou Maternal and Child Health Hospital Affiliated to Medical College of Yangzhou University, Yangzhou, Jiangsu 225001, China; 4 Department of Anesthesia and Perioperative Medicine, the First Affiliated Hospital with Nanjing Medical University, Nanjing, Jiangsu 210029, China; 5 Department of Rehabilitation, the First Affiliated Hospital of Nanjing Medical University, Nanjing, Jiangsu 210029, China

**Keywords:** wound healing, remote neuromuscular electrical stimulation, efferocytosis, MDK, LRP1

## Abstract

Neuromuscular electrical stimulation (NMES) is a well-established therapeutic approach for chronic wounds. Conventionally, NMES involves direct electrode contact with wounds or adjacent healthy skin; however, it is limited by the need for wound exposure and by increased pain. Our preliminary study demonstrated the innovative application of remote NMES (rNMES) to the skeletal muscle of the distal calf, which showed the potential to accelerate wound healing in remote areas. rNMES was effective in human clinical trials in our previous work, although the underlying mechanisms remain unclear. As rNMES is often used to stimulate muscle contraction in long-term bedridden patients, we analyzed data from the Gene Expression Omnibus (GEO) database and found that exercise promotes midkine (MDK) expression in muscle. MDK is a small secreted heparin-binding protein that interacts with multiple cell surface receptors to promote growth. In the present study, we found that MDK significantly enhanced macrophage efferocytosis in a low-density lipoprotein receptor-related protein 1 (LRP1)-dependent manner. Our findings demonstrate that rNMES upregulates MDK expression in skeletal muscles through the AMPK-ERK axis, facilitating its delivery to wounds through the circulatory system and promoting LRP1-mediated efferocytosis of apoptotic cells, thereby expediting wound healing.

## Introduction

Chronic refractory wounds, such as diabetic wounds, are characterized by their inability to achieve anatomical and functional integrity within predictable timeframes and stand as formidable challenges^[1]^. These wounds impose a substantial burden on patients. Thus, there is an urgent need to explore novel and efficacious therapeutic avenues capable of not only managing wound infections and chronic inflammatory reactions but also expediting the overall wound-healing process. Wound healing progresses through four distinct but intricately interconnected phases: hemostasis, inflammation, proliferation, and remodeling^[2]^. Chronic wounds, however, tend to dwell in the inflammatory phase for extended durations. This prolonged inflammation hinders the progression of wound repair. Hence, strategies aimed at ameliorating this persistent inflammatory state hold promise as potential treatments for chronic refractory wounds.

Traditional electrical stimulation therapy, a commonly employed modality in clinical practice to facilitate wound healing, presents various modalities, including unidirectional and bidirectional currents^[3]^. Unidirectional electrical stimulation involves the placement of the anode on the healthy skin surrounding the wound, with the cathode placed at the center of the wound. However, prolonged use induces thermal effects and may potentially harm the skin. Electrodes with bidirectional current are usually placed on the healthy skin on both sides of the wound. Nevertheless, this approach proves less effective for larger wounds and may inadvertently induce muscle contractions near the wound site, causing discomfort and additional pain.

Neuromuscular electrical stimulation (NMES) therapy has been shown to rapidly alleviate inflammation and promote wound healing^[4]^. In our preliminary research, we unexpectedly found that NMES applied to the distal skeletal muscles in the calves accelerated the healing of dorsal wounds. Despite this intriguing observation, the precise underlying mechanism remains elusive.

The most direct biological effect triggered by electrical stimulation is skeletal muscle contraction. Thus, it is often employed in patients who are bedridden for an extended period to simulate muscle contraction. Extensive research has demonstrated that skeletal muscle contractions, following physical exercise, trigger the release of numerous growth factors. Midkine (MDK), also known as neurite growth-promoting factor 2, operates as an autocrine growth factor with heparin-binding properties. Notably, elevated MDK levels following cerebral infarction or spinal cord injuries confer neuroprotective effects by inhibiting neuronal apoptosis^[5]^. MDK has also been reported to promote wound healing in diabetic rat models^[6]^. Furthermore, MDK plays a pivotal role in the synthesis of collagens and glycosaminoglycans, actively participating in tissue repair processes^[7]^. Notably, the association between MDK and electrical stimulation in the context of tissue repair has not yet been explored.

Low-density lipoprotein receptor-related protein 1 (LRP1) has been identified as a binding partner of MDK, exhibiting the highest affinity^[8]^. LRP1 serves as a critical mediator of macrophage efferocytosis, facilitating the clearance of apoptotic cells and, in turn, dampening inflammation^[9]^. Lung-resident macrophages phagocytose apoptotic neutrophils through LRP1 to resolve inflammation^[10]^.

In the current study, we aimed to elucidate the mechanism by which MDK is produced in skeletal muscle tissue. Furthermore, we investigated how MDK enhances efferocytosis, thereby promoting the clearance of apoptotic cells and mitigating inflammation by upregulating macrophage LRP1 expression. Our research endeavored to shed light on these intricate processes and their collective contributions to the acceleration of wound repair.

## Materials and methods

### Animals and excision wound model

Adult male Institute of Cancer Research (ICR) and C57BL/6 mice (20–25 g in weight) were provided by the Model Animal Research Center of Nanjing University, Nanjing, China. ICR mice were anesthetized with isoflurane and depilated. The wound area was disinfected with 75% ethanol. A puncher was used to create a circular full-thickness skin wound with a diameter of 1 cm to establish the full-thickness excision wound model. Photographs of the wounds were taken daily.

All procedures were strictly performed in accordance with the regulations of the ethics committee of the International Association for the Study of Pain and the Guide for the Care and Use of Laboratory Animals (The Ministry of Science and Technology of China, 2006). All animal experiments were approved by the Nanjing Medical University Animal Care and Use Committee and were designed to minimize suffering and the number of animals used (Approval No. IACUC-230513).

### Hematoxylin and eosin (H&E), Masson's trichrome, and TUNEL staining

Wound samples from mice were immersed in 4% paraformaldehyde for 4 h and transferred to 75% ethanol. Then, the samples were dehydrated through a series of alcohol gradients and embedded in paraffin wax blocks. Skin sections were dewaxed in xylene, rehydrated through decreasing concentrations of ethanol, and washed in phosphate-buffered saline (PBS; pH 7.4). The sections were stained with either hematoxylin and eosin (Cat. #C0105S, Beyotime, Shanghai, China), Masson's trichrome (Cat. #C0163S, Beyotime), or the Colorimetric TUNEL Apoptosis Assay Kit (Cat. #C1091, Beyotime). After staining, the sections were dehydrated through increasing concentrations of ethanol and xylene. Finally, images were captured using a Leica DM2500 microscope (Leica Microsystems, Wetzlar, Germany).

### Immunofluorescence staining

After antigen retrieval, wound sections were blocked with 10% donkey serum at room temperature for 1 h. The sections were then incubated with anti-F4/80 (1∶200, Cat. #sc-377009, Santa Cruz, CA, USA), anti-iNOS (1∶200, Cat. #ab178945, Abcam, Cambridge, UK), and anti-CD206 (1∶250, Cat. #ab64693, Abcam) overnight at 4 ℃. The secondary antibodies used were as follows: Alexa Fluor 594-conjugated donkey anti-mouse (1∶300, Cat. #715-585-150, Jackson ImmunoResearch, West Grove, PA, USA) and Alexa Fluor 488-conjugated donkey anti-rat (1∶300, Cat. #712-025-150, Jackson ImmunoResearch), which were incubated with the sections for 2 h at room temperature. After being washed three times with PBS, the samples were imaged using a Leica DM2500 microscope (Leica Microsystems).

### Quantification of neutrophil extracellular traps (NETs) in plasma

Mouse plasma was collected from whole blood by centrifugation at 1500 *g* for 5 min. NETs in plasma were quantified according to the manufacturer's instructions using the Quant-iT PicoGreen dsDNA Assay kit (Cat. #P11496, Invitrogen, Carlsbad, CA, USA). The fluorescence intensity was measured using a microplate reader (Multiskan FC, Thermo Fisher, Waltham, MA, USA).

### Isolation and cultivation of bone marrow-derived macrophages (BMDMs) and neutrophils

The isolation and culture of BMDMs and neutrophils were previously described in our published work^[11–12]^. Bone marrow cells were isolated from the femurs and tibias of C57BL/6J mice under sterile conditions. The cells were cultured in DMEM supplemented with 10% fetal bovine serum, 1% penicillin/streptomycin, and 10% L929 cell-conditioned medium. Cultures were maintained at 37 ℃ in a humidified incubator with 5% CO_2_. Fresh medium was replaced every 2–3 days. On day 7, differentiated BMDMs were harvested and plated for downstream experiments.

### Endocytosis of apoptotic neutrophils by BMDMs

BMDMs were pretreated with interferon-γ (IFN-γ; 50 ng/mL) and lipopolysaccharide (LPS; 100 ng/mL) for more than 24 h. Neutrophils were exposed to UV light for 15 min to induce apoptosis, followed by incubation with BCECF (1∶1000) at 37 ℃ for 30 min. Neutrophils were centrifuged at 1000 *g* for 8 min, resuspended in PBS, and added to the BMDMs. LDL receptor-related protein-associated protein (LRPAP; Cat. #HY-P76478A, MedChemExpress, Monmouth Junction, NJ, USA) was used as an LRP1 inhibitor. The samples were incubated at 37 ℃ for 15 min to allow phagocytosis. Non-phagocytosed apoptotic neutrophils were removed by rinsing with cold PBS. The proportion of phagocytosis-positive cells was determined using a Leica DM2500 microscope (Leica Microsystems).

### Gene ontology (GO) and pathway enrichment analysis

The mRNA profiles of human skeletal muscle (GSE117070) based on the GPL570 platform were downloaded from the Gene Expression Omnibus (GEO) database (https://www.ncbi.nlm.nih.gov/geo). The differentially expressed genes were identified using GEO2R (https://www.ncbi.nlm.nih.gov/geo/geo2r), with the criteria of |log_2_(fold change)| > 0.1 and *P* < 0.05. For pathway enrichment analysis, the "clusterProfiler" package in R was used to identify enriched pathways. Enriched GO terms and Kyoto Encyclopedia of Genes and Genomes (KEGG) pathways with a Benjamini-Hochberg adjusted *P*-value < 0.05 were selected.

### Electrical stimulation of C2C12 myoblast cells

C2C12 cells were induced to differentiate in DMEM containing 2% horse serum and 1% penicillin/streptomycin for 5 days. The electrode pads were fixed to the inner sides of the Petri dish, ensuring that they were always in contact with the medium. The Petri dish was fixed in the incubator, and electrical stimulation was initiated. Compound C (Cat. #HY-13418A, MedChemExpress) was used to inhibit AMPK, and SCH772984 (Cat. #S7101, Selleck Chemicals, Houston, TX, USA) was used to inhibit ERK1/2.

### Western blotting analysis

Samples (sciatic nerve and muscle tissues from mice) were collected, washed with PBS, and lysed in RIPA lysis buffer (Beyotime). Protein concentrations were measured by the BCA Protein Assay (Thermo Fisher). For cell culture supernatants, proteins were extracted using a methanol–chloroform precipitation method. Briefly, methanol and chloroform were added to the supernatants for protein precipitation, followed by centrifugation and removal of the supernatant. The protein pellet was further purified with chloroform, air-dried, and dissolved in 1× SDS loading buffer for Western blotting analysis. Sample lysates were then separated by SDS-PAGE and electrophoretically transferred onto polyvinylidene fluoride membranes (Millipore Corp., Bedford, MA, USA). The membranes were blocked with 10% nonfat milk in TBST at room temperature for 2 h and then probed with primary antibodies at 4 ℃ overnight. The primary antibodies used were MDK (1∶750, Cat. #A0251, Abclonal, Wuhan, China), LRP1 (1∶10000, Cat. #ab31828, Abcam), MMP-9 (1∶1500, Cat. #ab76003, Abcam), p-p38 (1∶1000, Cat. #ab195049, Abcam), β-actin (1∶10000, Cat. #AC026, Abclonal), Albumin (1∶20000, Cat. #A24161, Abclonal), p-AMPK (1∶1000, Cat. #2535, Cell Signaling Technology, Danvers, MA, USA), p-ERK1/2 (1∶1000, Cat. #ab17942, Abcam), p-JNK (1∶1000, Cat. #ab307802, Abcam), protein disulfide isomerase (PDI; 1∶1000, Cat. #ab2792, Abcam), p-ADAM17 (1∶1000, Cat. #ab182630, Abcam), IL-10 (1∶1000, Cat. #60269-1-Ig, Proteintech, Wuhan, Hubei, China), and TGF-β (1∶1000, Cat. #3711, Cell Signaling Technology). The membranes were then incubated with secondary antibodies from Sigma (St. Louis, MO, USA) for 2 h. Protein bands were visualized using a Molecular Imager (Gel Doc XR, 170-8170) and analyzed using Quantity One 4.6.5 (Bio-Rad Laboratories, Berkeley, CA, USA).

### Reverse transcription-quantitative PCR (RT-qPCR)

Total RNA from RAW264.7 cells was isolated using TRIzol reagent (Invitrogen Life Technologies) according to the manufacturer's instructions. The isolated RNA was reverse-transcribed into cDNA using the PrimeScript RT Reagent Kit (Takara, Shiga, Japan) following standard protocols. Quantitative PCR (qPCR) was performed using synthetic primers and SYBR Green (Takara) on a QuantStudio 5 Real-Time PCR Detection System (Thermo Fisher Scientific). The relative expression levels of *LRP1* were quantified using the 2^−ΔΔCt^ method after normalization to the reference gene β-actin. The following primers were used: β-actin, 5′-GGCTGTATTCCCCTCCATCG-3′ (forward) and 5′-CCAGTTGGTAACAATGCCATGT-3′ (reverse); *LRP1*, 5′-ACTATGGATGCCCCTAAAACTTG-3′ (forward) and 5′-GCAATCTCTTTCACCGTCACA-3′ (reverse).

### Statistical analysis

All statistical analyses were performed using GraphPad Prism 8 software (GraphPad Software, San Diego, CA, USA). Data were analyzed using one-way or two-way ANOVA with Bonferroni post hoc test, as indicated in the figure legends. Data were expressed as the mean ± standard error of the mean. A *P*-value < 0.05 was considered statistically significant.

## Results

### Electrical stimulation of skeletal muscle accelerated skin wound healing

**Figure 1:**
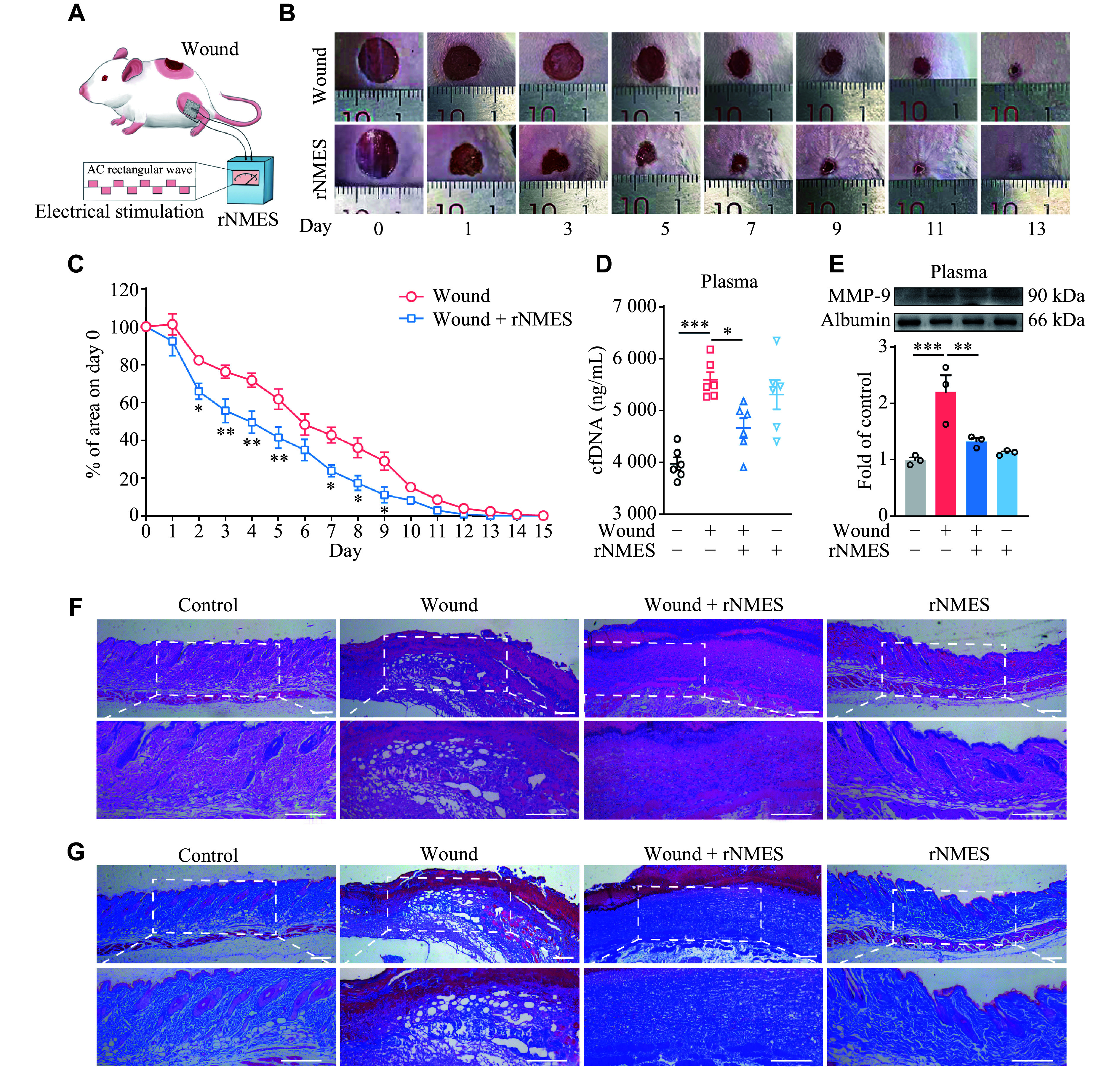
Acceleration of skin wound healing by electrical stimulation of skeletal muscle. A: Schematic depiction of animal experiments. A wound model was established on the back of the Institute of Cancer Research mice. Electrical stimulation patches were placed on the inner and outer sides below the knee. Stimulation parameters consisted of alternating current (AC) rectangular waves (3 mA, 100 Hz, 500 μs pulse width) administered for 1 h/day. B: Representative images of the wound on days 0, 1, 3, 5, 7, 9, 11, and 13. C: Changes in wound area in both groups over 15 days (*n* = 8). D–G: Mice were sacrificed on day 4. (D) The content of cfDNA in plasma (*n* = 6). (E) Western blotting and quantitative analysis of MMP-9 protein level in plasma (*n* = 3). (F) Hematoxylin and eosin staining of the wound. (G) Masson's trichrome staining of the wound. Scale bars, 200 μm. All data are presented as mean ± standard error of the mean.^ *^P < 0.05, ^**^P < 0.01, and ^***^P < 0.001 by two-way ANOVA (C) and one-way ANOVA followed by Tukey's multiple comparisons test (D and E). Abbreviations: cfDNA, circulating free DNA; MMP-9, matrix metalloproteinase 9; NETs, neutrophil extracellular traps; rNMES, remote neuromuscular electrical stimulation.

A wound healing model was established on the mouse dorsal region (***Fig. 1A***). Remote NMES (rNMES) was precisely applied to the calf muscles. Remarkably, the rNMES group exhibited a significantly shortened wound healing phase compared with the control group (***Fig. 1B*** and ***1C***). In the initial stages of wound healing, neutrophils rapidly recruit to the site and release their DNA to form NETs, which capture bacteria, initiate the inflammatory response, and limit infection^[13]^. Concurrently, matrix metalloproteinase-9 (MMP-9) degrades the extracellular matrix, mediating vascular leakage and promoting the maturation of pro-inflammatory factors. The persistent presence of NETs and MMP-9 leads to delayed wound healing^[13]^. Following rNMES, both circulating free DNA (cfDNA) and MMP-9 levels in plasma were significantly reduced (***Fig. 1D*** and ***1E***).

Histopathological examination of the wounds showed that in both the control and rNMES alone groups, epithelial cells were morphologically normal. The collagen fibers in the dermis were more neatly arranged in these groups, indicating a relatively normal tissue state. In contrast, the wound model group displayed minimal granulation tissue regeneration, characterized by reduced collagen deposition and a loose, disorganized structure. Notably, rNMES treatment enhanced granulation tissue formation compared with that in the wound model, featuring abundant fiber proliferation, compact cellular arrangement, and increased collagen synthesis (***Fig. 1F*** and ***1G***).

### Indirect enhancement of efferocytosis through electrical stimulation

**Figure 2:**
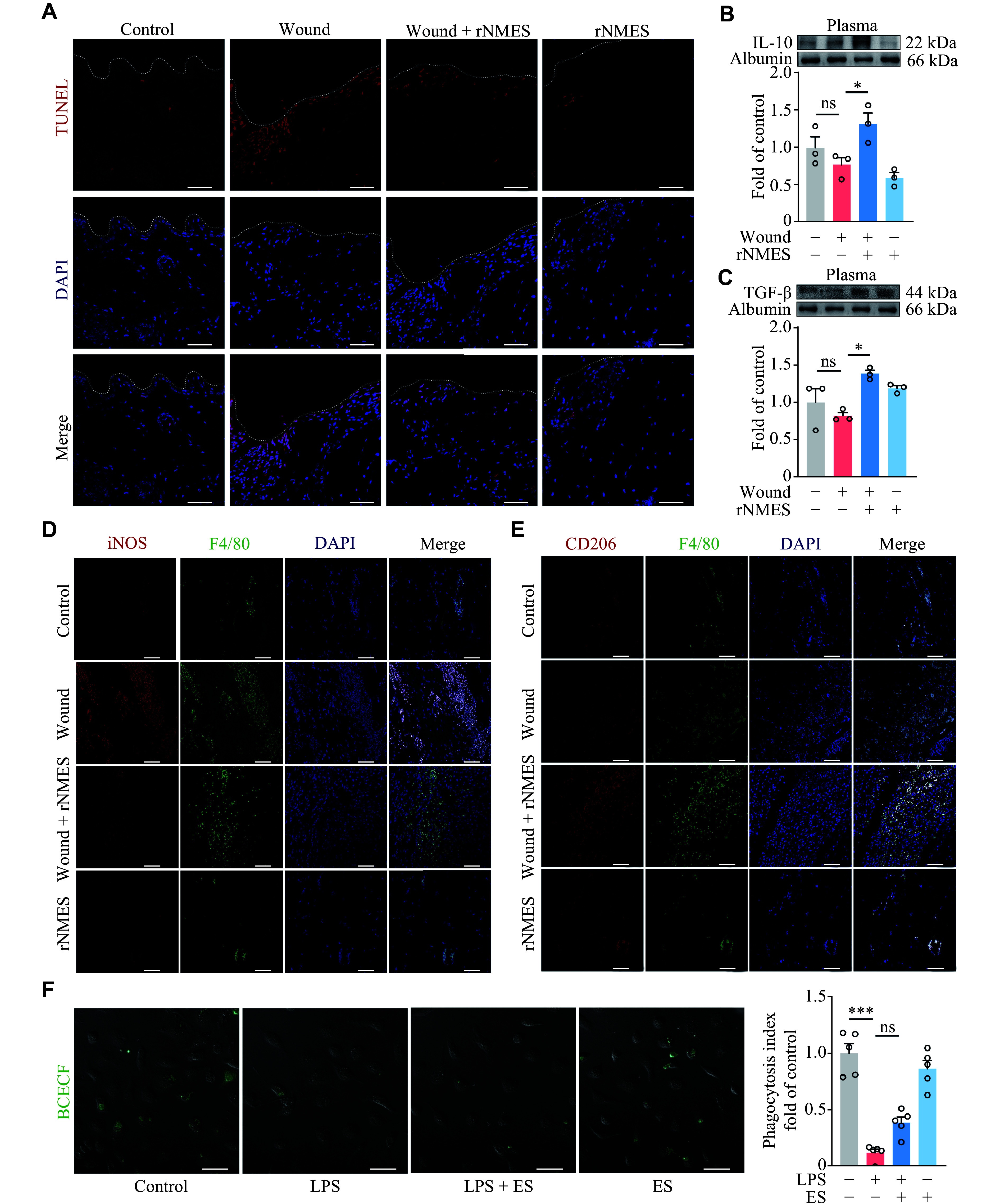
Indirect enhancement of efferocytosis through electrical stimulation. Alternating current rectangular wave, 3 mA, 100 Hz, pulse width 500 μs, 1 h/day. Mice were sacrificed on day 4. A: TUNEL staining of the wound sections. B and C: Western blotting and quantitative analysis of IL-10 (B) and TGF-β (C) levels in the plasma (*n* = 3). D and E: Immunofluorescence staining of wound sections showing the localization and expression levels of iNOS (M1 marker; red) and F4/80 (green) (D) and CD206 (M2 marker; red) and F4/80 (green) (E). F: Fluorescence staining of apoptotic neutrophils phagocytosed by BMDMs. BMDMs were treated with LPS (1 μg/mL) for 23 h with or without direct electrical stimulation (ES; 0.1 mA, 20 Hz). Apoptotic neutrophils (green) were added 1 h later. The ratio of phagocytosis-positive cells was quantified using confocal microscopy (*n* = 5). All data are presented as mean ± standard error of the mean. ^*^P < 0.05 and ^***^P < 0.001 by one-way ANOVA followed by Tukey's multiple comparisons test (B, C, and F). Scale bars, 50 μm. Abbreviations: IL-10, interleukin-10; LPS, lipopolysaccharide; NETs, neutrophil extracellular traps; ns, not significant; rNMES, remote neuromuscular electrical stimulation; TGF-β, transforming growth factor-β.

A profusion of apoptotic cells is commonly found within wounds, and their swift clearance by macrophages is essential for preventing prolonged inflammation^[14]^. TUNEL staining of the wounds revealed a substantial presence of apoptotic cells in the model group (***Fig. 2A***). rNMES resulted in a significant reduction in apoptotic cells, indicating enhanced macrophage-mediated phagocytosis within the wound. This process, known as efferocytosis, is pivotal in driving macrophages toward an anti-inflammatory phenotype (M2) from their pro-inflammatory counterpart (M1). This transition is characterized by the expression of anti-inflammatory factors, including transforming growth factor-β (TGF-β) and interleukin-10 (IL-10)^[15]^. We examined macrophage phenotypes in the wounds by immunofluorescence staining. In the model group, macrophages were predominantly M1, with only a few M2 (***Fig. 2D*** and ***2E***). rNMES expedited the shift of macrophages from M1 to M2, concurrently with elevated levels of TGF-β and IL-10 in plasma (***Fig. 2B*** and ***2C***).

We further investigated the effect of electrical stimulation on the phagocytosis of apoptotic neutrophils by BMDMs. Confocal microscopy was used to count the phagocytosis-positive cells. The results showed that BMDMs demonstrated robust phagocytic activity, which was attenuated by LPS (***Fig. 2F***). However, direct electrical stimulation of BMDMs did not improve phagocytic function. These findings collectively suggest that rNMES may indirectly enhance phagocytic activity.

### Identification of MDK and its role in enhanced efferocytosis due to electrical stimulation

**Figure 3:**
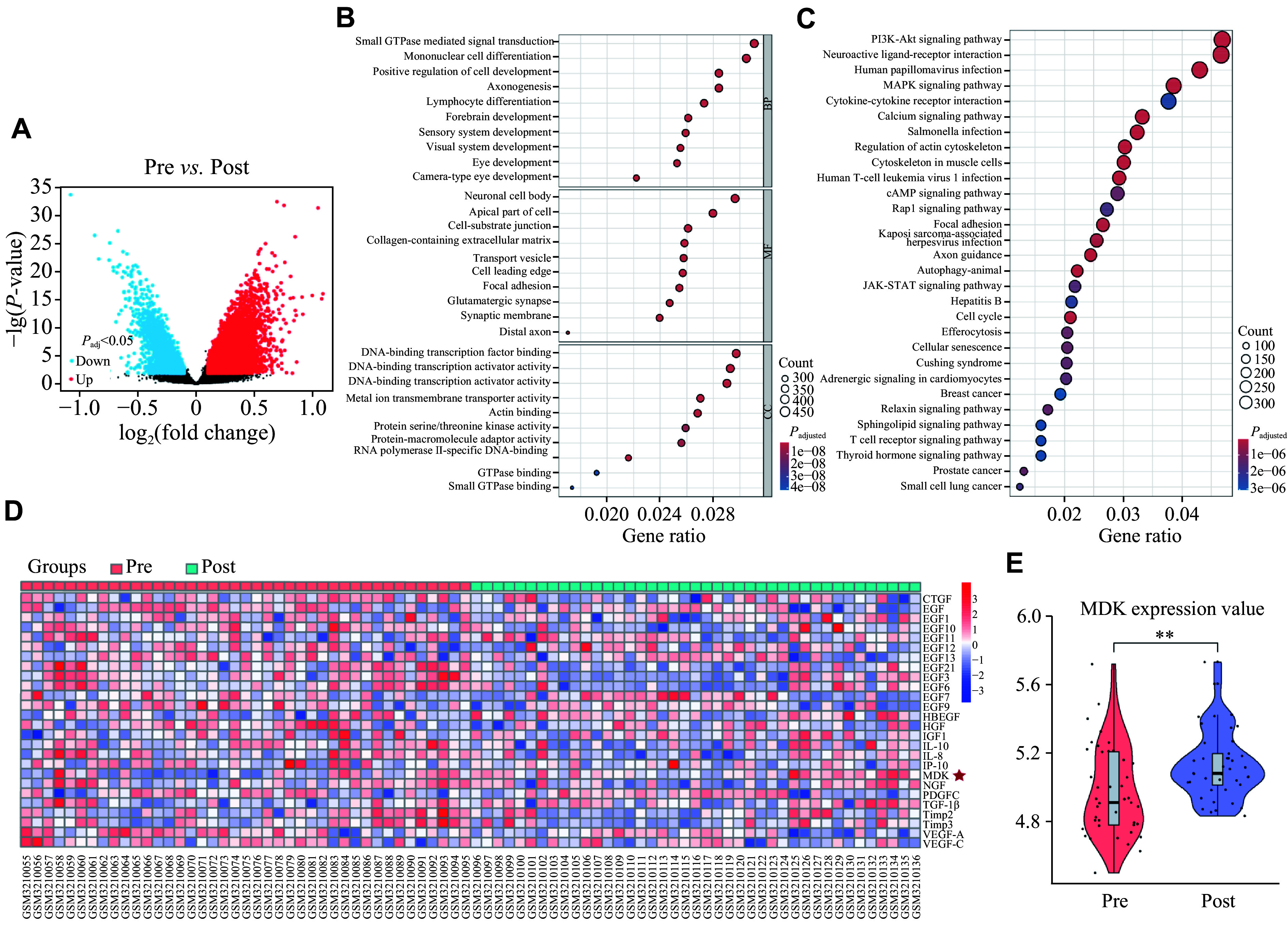
Bioinformatics analysis of human skeletal muscle after training. A: Volcano plot showing differentially expressed genes in the dataset GSE117070. B and C: GO and KEGG enrichment analysis. D: Heatmap of potential muscle-released growth factors. E: The expression of MDK in human skeletal muscle. All data are presented as mean ± standard error of the mean. ^**^P < 0.01 by two-tailed Student's *t*-tests (E). Abbreviations: BP, biological process; CC, cellular component; MDK, midkine; MF, molecular function.

**Figure 4:**
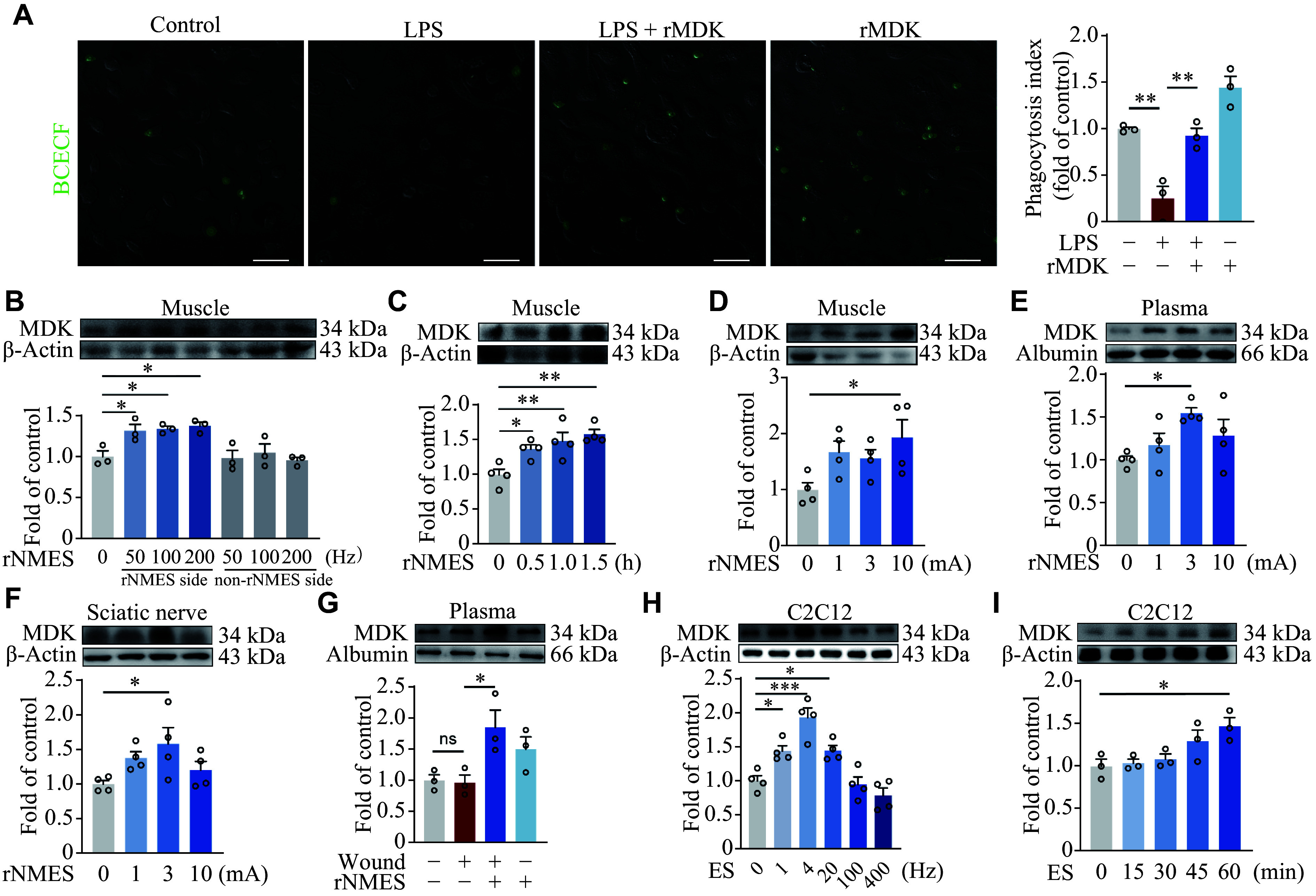
Upregulation of MDK and its role in enhanced efferocytosis due to electrical stimulation. A: Bone marrow-derived macrophages (BMDMs) were treated with LPS (1 μg/mL) for 12 h. Apoptotic neutrophils (green) were added to BMDMs, with or without rMDK (200 ng/mL) for 12 h. Confocal microscopy was used to quantify the phagocytosis index. Scale bars, 50 μm. B: rNMES (1 h, 3 mA, frequency as indicated) was applied for 3 days. MDK protein levels in both sides of the skeletal muscle were detected. C: rNMES (100 Hz, 3 mA, duration as indicated) was applied for 3 days. MDK protein levels in the skeletal muscle were detected. D–F: rNMES (100 Hz, 1 h, current as indicated) was applied for 3 days. MDK protein levels in skeletal muscle (D), plasma (E), and sciatic nerve (F) were detected. G: Mice were sacrificed on day 4 after the establishment of the wound model, with or without rNMES (1 h, 3 mA, 100 Hz). MDK protein levels in the plasma were detected. H and I: Electrical stimulation (ES) was applied to C2C12 cells for 1 h at 0.1 mA (frequency as indicated; H) or at 4 Hz at 0.1 mA (duration as indicated; I). MDK protein levels in the culture supernatant were detected. All data are presented as mean ± standard error of the mean. ^*^P < 0.05, ^**^P < 0.01, and ^***^P < 0.001 by one-way ANOVA followed by Tukey's multiple comparisons test (A–I). Abbreviations: LPS, lipopolysaccharide; MDK, midkine; ns, not significant; rMDK, recombinant MDK; rNMES, remote neuromuscular electrical stimulation.

Given that electrical stimulation failed to directly enhance efferocytosis, we hypothesized that rNMES might facilitate the release of molecules conducive to this process. rNMES induces skeletal muscle contraction, thus rendering it an effective approach for simulating exercise in long-term bedridden patients. We investigated whether exercise could trigger the production of certain growth factors in muscles, thereby promoting wound healing. To this end, we analyzed the human skeletal muscle gene expression dataset (GSE117070), which contains muscle biopsy data from participants following exercise training. Specifically, this dataset includes gene expression profiles from the vastus lateralis muscle, collected after physical exercise, allowing us to identify potential growth factors released in response to muscle contraction and exercise (***Fig. 3A***). The GO and KEGG analyses revealed significant enrichment of biological processes and pathways such as "small GTPase-mediated signal transduction," "regulation of the actin cytoskeleton," and "cytoskeleton in muscle cells" among differentially expressed genes in muscle tissue following exercise, suggesting that vesicle transport and secretion in muscles are significantly enhanced after exercise (***Fig. 3B*** and ***3C***). Subsequently, we identified several potential growth factors that may be conducive to wound healing and constructed a heatmap (***Fig. 3D***). The majority of growth factors exhibited no significant alterations after exercise, whereas MDK expression in muscle tissue following training was significantly elevated (***Fig. 3E***). MDK has been recognized for its tissue repair-promoting properties^[16]^. Thus, we hypothesized that MDK may be the factor released by skeletal muscle in response to rNMES that promotes efferocytosis in wound healing. We treated BMDMs with recombinant MDK (rMDK) to validate this hypothesis and found that rMDK significantly ameliorated LPS-induced BMDM efferocytosis dysfunction (***Fig. 4A***; ***Supplementary Videos 1–4***).

Subsequently, we measured MDK protein levels in the plasma and skeletal muscle of mice following rNMES treatment with varying stimulation parameters. As shown in ***Fig. 4B***–***4E*** and ***4G***, the highest MDK expression in skeletal muscle was observed at a current intensity of 10 mA, while the highest circulating MDK level was detected at 3 mA. A stimulation duration of 1 h increased skeletal muscle MDK expression by approximately 1.5-fold. No significant differences in skeletal muscle MDK levels were observed across stimulation frequencies ranging from 50 to 200 Hz. Notably, the elevation in MDK expression was limited to the stimulated side of the skeletal muscle. Based on these findings, the optimal rNMES parameters selected for subsequent experiments were 3 mA, 100 Hz, and 1 h stimulation. We also found elevated MDK levels in the sciatic nerve after rNMES (***Fig. 4F***). Additionally, C2C12 mouse myoblasts were used to validate the effect of rNMES, and ***Fig. 4H*** and ***4I*** show MDK release in response to electrical stimulation in the supernatant of C2C12 cell cultures.

### MDK upregulated LRP1 to improve efferocytosis through the PDI-ADAM17 axis

**Figure 5:**
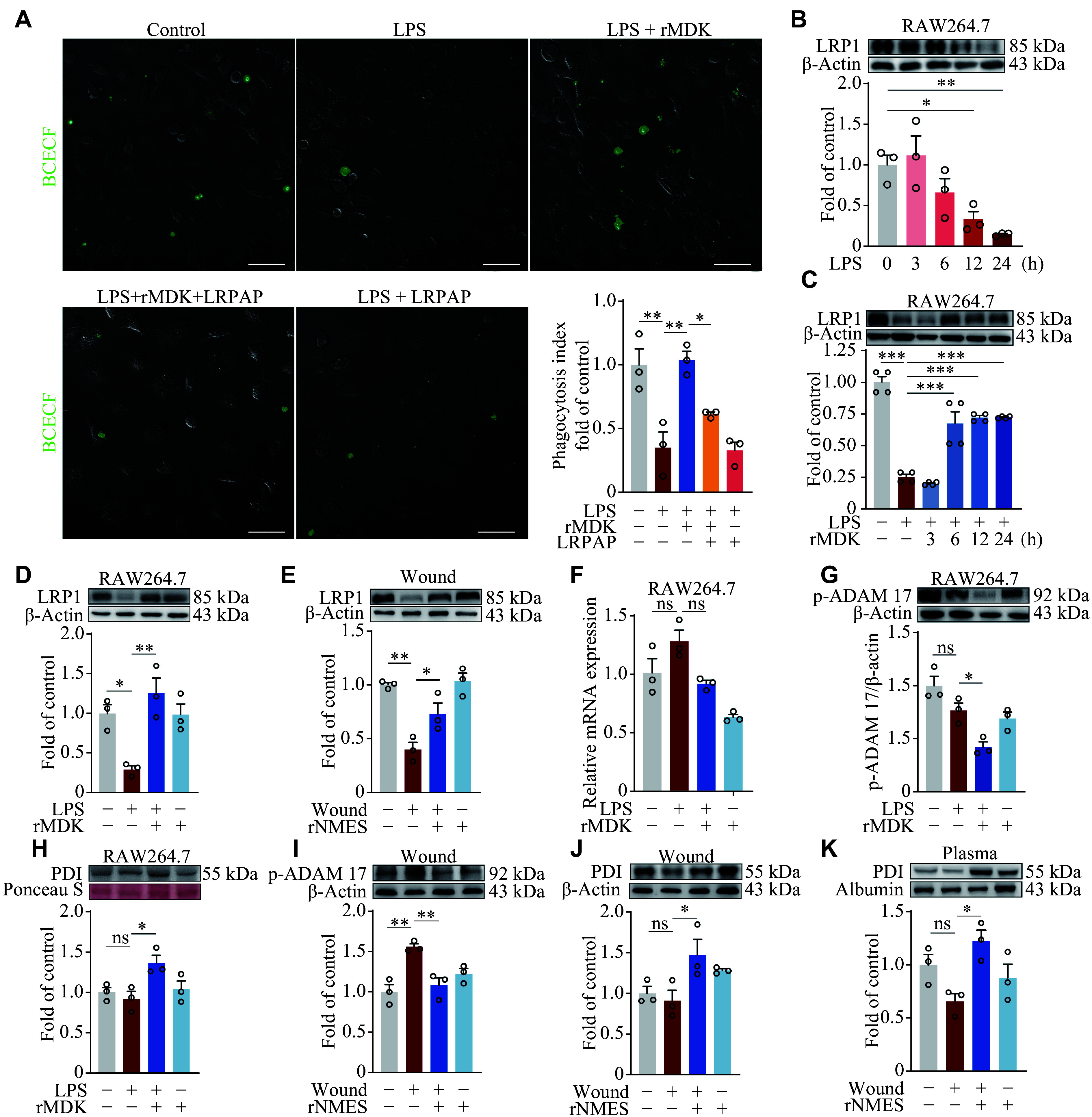
MDK upregulated LRP1 to improve efferocytosis through the PDI-ADAM17 axis. A: Bone marrow-derived macrophages (BMDMs) were treated with LPS (1 μg/mL) for 12 h. Apoptotic neutrophils (green) were added to BMDMs, with or without rMDK (200 ng/mL) for 12 h. LRPAP (50 μmol/L) was applied 0.5 h before rMDK. Confocal microscopy was used to quantify the phagocytosis index. Scale bars, 50 μm. B–D: LRP1 protein levels in RAW264.7 cells. Cells were treated with LPS (1 μg/mL) for 0, 3, 6, 12, 24 h (B), with LPS (1 μg/mL) for 24 h and rMDK for 3, 6, 12, 24 h (C), or with LPS (1 μg/mL) for 24 h with or without rMDK (200 ng/mL) for 12 h (D). E: LRP1 protein levels in the wound model. F and G: *Lrp1* mRNA (F) and p-ADAM17 protein (G) levels in RAW264.7 cells treated as in (D). H: PDI protein levels in the supernatant of RAW264.7 cell cultures. I and J: p-ADAM17 (I) and PDI (J) protein levels in the wound. K: PDI protein levels in the plasma. Protein and mRNA levels were analyzed by Western blotting and reverse transcription-quantitative PCR, respectively. All data are presented as mean ± standard error of the mean. ^*^P < 0.05, ^**^P < 0.01, and ^***^P < 0.001 by one-way ANOVA followed by Tukey's multiple comparisons test (A–K). Abbreviations: LPS, lipopolysaccharide; LRP1, LDL receptor-related protein 1; LRPAP, LDL receptor-related protein-associated protein; ns, not significant; p-ADAM17, phospho-a disintegrin and metalloproteinase domain-containing protein 17; PDI, protein disulfide isomerase; rMDK, recombinant midkine; rNMES, remote neuromuscular electrical stimulation.

Among the cell surface receptors identified as binding to MDK, LRP1 serves as an efferocytosis receptor^[17]^. LRPAP, an inhibitor of LRP1, reversed the protective effect of rMDK against LPS-impaired efferocytosis in BMDMs (***Fig. 5A***). These results suggest that MDK may promote efferocytosis through LRP1. Subsequently, we detected LRP1 protein levels by Western blotting in RAW264.7 cells treated with LPS for various durations and found that the levels decreased rapidly as the duration of LPS treatment increased (***Fig. 5B***). Notably, rMDK significantly restored the LPS-induced downregulation of LRP1 protein levels when applied for over 6 h (***Fig. 5C***). ***Fig. 5D*** depicts LRP1 levels after 24 h of LPS treatment, with or without rMDK for 12 h. Additionally, LRP1 levels were significantly decreased in wound model mice, compared with the controls but were significantly restored following rNMES treatment (***Fig. 5E***).

PCR analysis revealed that rMDK did not significantly upregulate *Lrp1* mRNA levels (***Fig. 5F***), implying that the effect of rMDK on LRP1 occurs through post-transcriptional regulation or inhibition of its degradation. As a cell surface receptor, LRP1 is susceptible to shedding by a disintegrin and metalloproteinase 17 (ADAM17)^[18]^. PDI serves as an inhibitor of ADAM17. PDI catalyzes the isomerization of the disulfide bond in the membrane-proximal domain of ADAM17, leading to a conformational transition from an open state to a closed state^[19]^. In the current study, we observed that rMDK markedly reduced ADAM17 phosphorylation and increased PDI levels in RAW264.7 cells following LPS stimulation (***Fig. 5G*** and ***5H***). In the wound model of mice, rNMES resulted in increased PDI levels but decreased p-ADAM17 levels (***Fig. 5I***–***5K***). These findings collectively underscore the role of rNMES in upregulating PDI to inhibit p-ADAM17 *via* MDK, thereby facilitating efferocytosis.

### Electrical stimulation upregulated MDK through the AMPK-ERK axis

**Figure 6:**
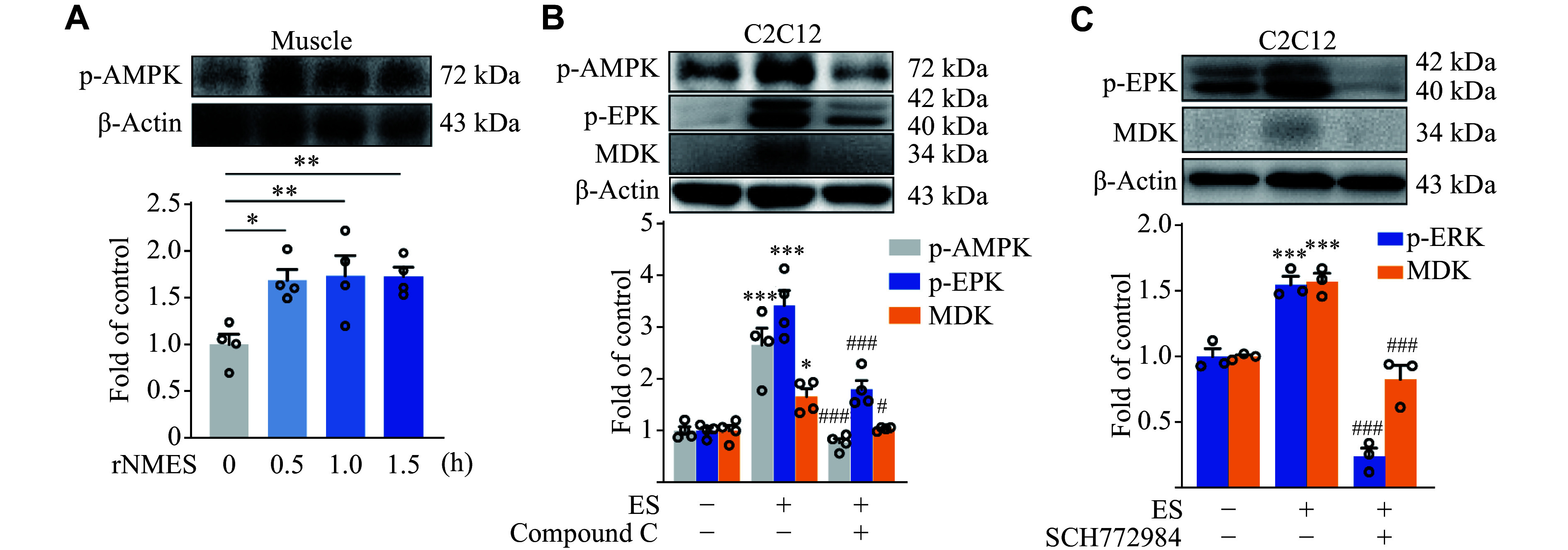
Electrical stimulation upregulated MDK through the AMPK-ERK axis. A: rNMES was applied for 3 days (100 Hz, 3 mA). MDK protein levels in the skeletal muscle. ^*^P < 0.05 and ^**^P < 0.01 *vs.* the 0 h group. B: Compound C (20 μmol/L) was used 15 min before ES (0.1 mA, 20 Hz, 1 h) to inhibit AMPK. p-AMPK and p-ERK protein levels in C2C12, and MDK protein levels in the supernatant of the cell cultures. C: SCH772984 (2 μmol/L) was used 30 min before ES (0.1 mA, 4 Hz, 1 h) to inhibit ERK. p-ERK protein levels in C2C12 and MDK protein levels in the supernatant of the cell cultures. Protein levels were analyzed by Western blotting. All data are presented as mean ± standard error of the mean. ^*^P < 0.05 and ^***^P < 0.001 *vs.* the control group, ^#^*P* < 0.05 and ^###^*P* < 0.001 *vs.* the ES group (B–C). Statistical analysis was performed using one-way ANOVA followed by Tukey's multiple comparisons test. Abbreviations: MDK, midkine; p-AMPK, phospho-AMP-activated protein kinase; p-ERK, phospho-extracellular signal-regulated kinase; rNMES, remote neuromuscular electrical stimulation; ES, electrical stimulation.

Previous research has underscored the beneficial effects of pulsed rNMES on wound healing, which are mediated through the TGF-β1/ERK/NF-κB signaling pathway^[20]^. Furthermore, electroacupuncture has been shown to alleviate ischemic stroke by upregulating the MDK and ERK signaling pathways^[21]^. Notably, rNMES has been shown to significantly activate AMPK following a brief 1-h application in C2C12 cells^[22]^. Consistently, our results demonstrated a significant elevation in phosphorylated AMPK levels within skeletal muscle following rNMES (***Fig. 6A***). Hence, it is reasonable to hypothesize that rNMES upregulates MDK expression in skeletal muscle by activating the AMPK-ERK-NF-κB signaling cascade. To validate this hypothesis, we treated C2C12 cells with electrical stimulation (0.1 mA, 20 Hz, 1 h) and observed significant activation of AMPK and ERK1/2 (***Fig. 6B***). Concurrently, a significant increase in MDK levels was detected in the supernatant. This increase was reversed by treatment with the AMPK inhibitor Compound C (***Fig. 6B***). Furthermore, the administration of the ERK1/2 inhibitor SCH772984 significantly attenuated the stimulatory effect of electrical stimulation, thereby highlighting that rNMES orchestrates the upregulation of skeletal muscle MDK through the AMPK-ERK axis (***Fig. 6C***). This intricate signaling pathway underlies a critical aspect of rNMES-mediated MDK elevation.

## Discussion

**Figure 7:**
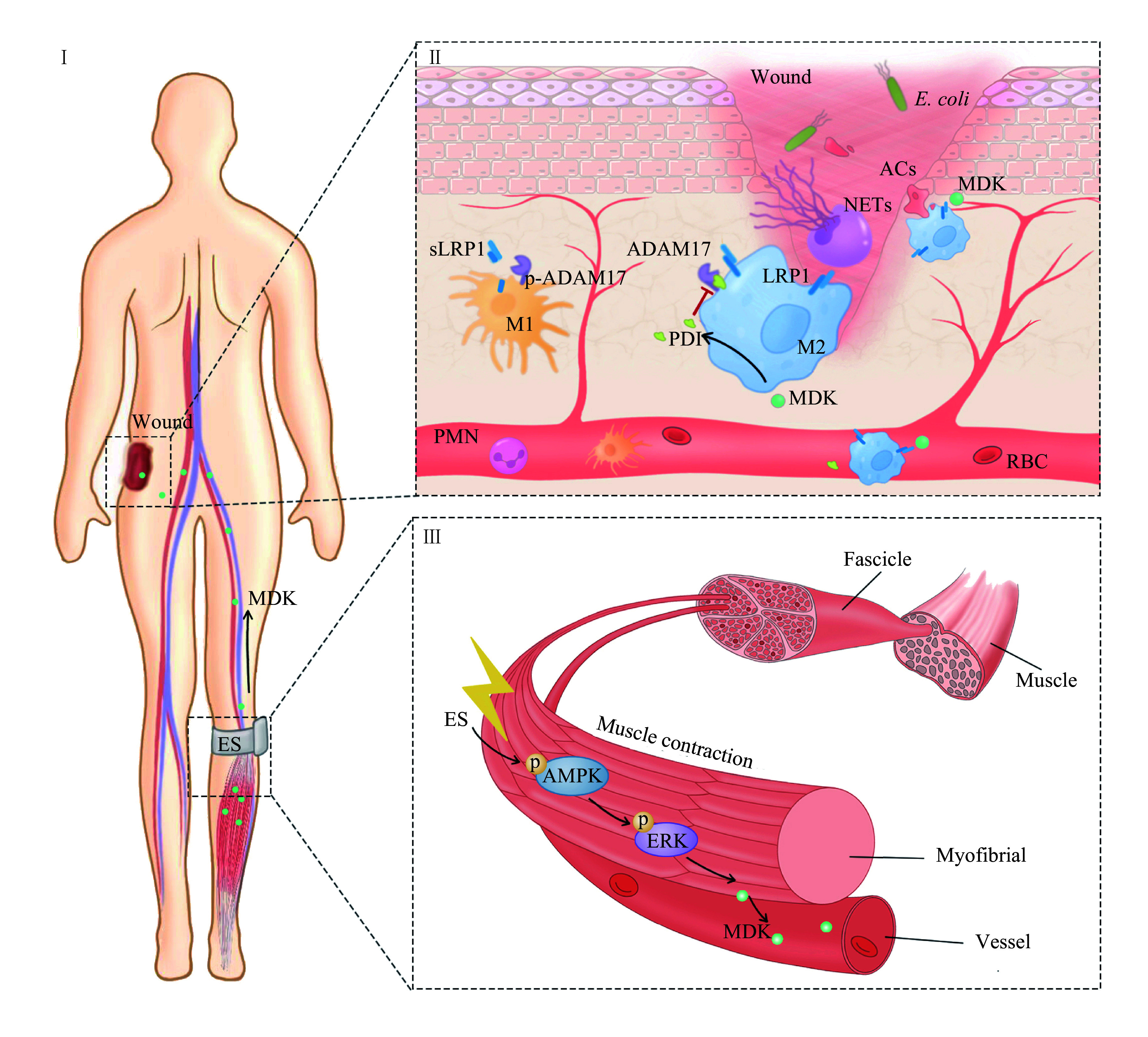
A schematic diagram showing the mechanism of rNMES in wound healing. rNMES induces MDK production in skeletal muscles through the AMPK-ERK axis. The secreted MDK then reaches the wound *via* blood circulation, enhances LRP1-mediated efferocytosis, and accelerates wound healing. Abbreviations: MDK, midkine; LRP1, low-density lipoprotein receptor-related protein 1; sLRP1, soluble LRP1; p-ADAM17, phospho-a disintegrin and metalloproteinase domain-containing protein 17; PDI, protein disulfide isomerase; p-AMPK, phospho-AMP-activated protein kinase; p-ERK, phospho-extracellular signal-regulated kinase; PMN, polymorphonuclear neutrophil; RBC, red blood cell; rNMES, remote neuromuscular electrical stimulation; NETs, neutrophil extracellular traps; ACs, apoptotic cells; *E. coli*, *Escherichia coli*.

This study yields the following key findings: (1) rNMES applied to skeletal muscle significantly accelerated the healing of distant skin wounds. This intervention reduced NETs and MMP-9 levels in the plasma. (2) rNMES promoted both the clearance of apoptotic cells by macrophages and M2 polarization. (3) rNMES, simulating exercise, increased MDK levels in both muscle tissue and the plasma of wounded mice. Subsequently, we demonstrated that MDK played a pivotal role in enhancing apoptotic cell phagocytosis by macrophages, a process known as efferocytosis. (4) This enhancement occurred through the upregulation of LRP1 in macrophages, which was facilitated by the PDI-ADAM17 axis, thereby promoting efficient wound healing. (5) Finally, rNMES upregulated MDK through the AMPK-ERK signaling pathway (***Fig. 7***).

Current clinical treatments for wounds include debridement, flap grafting, dressings, physiotherapy, compression therapy, and stem cell therapy^[23]^. However, each of these methods has its limitations and disadvantages. Electrical stimulation therapy is commonly used in rehabilitation and has demonstrated efficacy in promoting tissue repair across various conditions. In this context, we introduce rNMES as a novel application of electrical stimulation that specifically targets distal skeletal muscle to modulate the innate immune response within wounds. It is not limited by wound size and can be integrated with existing treatments without direct wound exposure. This technique leverages the body's innate mechanisms to enhance wound healing, similar to remote ischemic post-conditioning. Both techniques share the feature of indirectly modulating the distant environment, making them complementary to conventional treatments. Our recently published study demonstrated that rNMES therapy promoted wound healing in human clinical trials, with subsequent animal experiments conducted to elucidate the underlying molecular mechanisms^[24]^. Xing *et al*^[21]^ demonstrated that electroacupuncture stimulation of the Quchi (LI11) and Zusanli (ST36) acupoints upregulated MDK levels in the cerebral cortex and improved stroke outcomes, yet they did not elucidate the molecular mechanisms. In contrast, our study not only elucidated the mechanism by which rNMES induced MDK expression through the AMPK-ERK pathway but also unveiled MDK's ability to enhance efferocytosis through the LRP1 receptor, mediated by the PDI-ADAM17 axis, thereby accelerating wound healing.

MDK has been shown to play a critical role in the early stages of wound healing. As a stimulator of collagen and glycosaminoglycan synthesis, MDK has potential as a therapeutic agent for delayed wound healing^[7]^. Additionally, MDK has been shown to upregulate growth factors, such as platelet-derived growth factor (PDGF), TGF-β, and vascular endothelial growth factor (VEGF), in diabetic wounds^[6]^. Wound dressings with exogenously added MDK have been developed, but the underlying mechanism remains unclear. The N-terminal domain of MDK interacts with LRP1, driving neutrophil and macrophage trafficking^[25]^. This is consistent with our histological findings, as evidenced by the increased infiltration of inflammatory cells in rNMES-treated wounds. In the wound-healing process, the inflammatory phase functions as the primary defense against pathogens. Repair is compromised when immune cell recruitment is inadequate, as observed in Toll-like receptor 3 (*Tlr3*) knockout mice^[26]^. However, an uncontrolled, excessive inflammatory response can exacerbate tissue damage and delay the healing process^[27]^. Thus, a balanced immune response that adapts to local conditions, rapidly controlling infection and effectively clearing cellular debris, is essential for successful wound healing.

Normally, most neutrophils adhere to the blood crust and are subsequently shed, while others return to circulation, undergo apoptosis or necrosis, and are eventually cleared by macrophages through efferocytosis^[28]^. In chronic wounds, macrophages display inefficient phagocytosis and a persistent pro-inflammatory polarization state^[29]^. The immunomodulatory effects of rNMES on macrophages have been reported. In a rat amputation model, rNMES applied to the stump tissue was associated with a significant increase in the number of classically activated (M1) macrophages on day 3, followed by a gradual decrease, while alternatively activated (M2) macrophages showed higher levels on both days 3 and 7^[30]^. Our results also showed that rNMES reduced the number of M1 macrophages and increased the number of M2 macrophages in the wound. M2 macrophages produced IL-10 and TGF-β to promote epithelialization, fibroproliferation, and angiogenesis, while promptly removing apoptotic cells from the wound, providing a better environment for subsequent repair.

LRP1 on macrophages recognizes calreticulin on apoptotic cells and initiates efferocytosis^[31]^. *LRP1* knockdown leads to the upregulation of TLR4 expression^[32]^, thereby further intensifying the inflammatory response mediated by damage-associated molecular patterns (DAMPs) and pathogen-associated molecular patterns (PAMPs). We report for the first time that rNMES applied to skeletal muscle induces MDK production, which regulates LRP1 levels in wound macrophages. This effect is achieved by upregulating PDI to protect LRP1 from degradation by ADAM17.

This study demonstrates the therapeutic potential of rNMES for tissue repair and regeneration. Previous studies have highlighted the importance of immune modulation and angiogenesis in wound healing^[33–34]^. Given its ability to modulate macrophage functions and promote tissue remodeling, rNMES holds significant promise as a therapeutic intervention for chronic wounds, particularly in diabetic patients. Further research should optimize stimulation protocols and evaluate the clinical applicability of rNMES in broader medical contexts. Although our study showed good efficacy in the full-thickness excision wound model, further validation is needed in chronically infected and thermal injury models, which are more prevalent in elderly and diabetic populations. Furthermore, our study focused on the 4-day time point, where the greatest differences in wound healing were observed, with complete wound closure achieved by day 15 and no further recurrence. However, the current data do not address the long-term effects of rNMES on tissue remodeling, wound stability, or recurrence. Future studies should extend follow-up periods to evaluate these aspects, including tissue strength, scar formation, and immune response during the remodeling phase. These longer-term investigations will provide a more comprehensive understanding of the sustained effects of rNMES on wound healing.

Our results demonstrate that rNMES upregulates MDK in skeletal muscle through the AMPK-ERK pathway, thereby enhancing LRP1-dependent macrophage efferocytosis and promoting wound repair. These findings provide a novel theoretical foundation for rNMES therapy and expand its potential applications in wound healing.

In summary, our study demonstrates that rNMES induces the production of MDK in skeletal muscle, which is subsequently transported to the wound site and may enhance LRP1-mediated efferocytosis, contributing to accelerated wound healing. However, it is important to note that other factors, such as systemic inflammatory responses and muscle contraction-related mechanisms, may also influence the observed effects. Further investigations are needed to fully elucidate the complex network of factors involved in tissue repair and regeneration. Collectively, these results offer a promising therapeutic avenue for wound healing, pending further validation of the broader mechanistic context.

## SUPPLEMENTARY DATA

Supplementary data to this article can be found online.
