# Supplementary material for: Remote neuromuscular electrical stimulation upregulates MDK to enhance macrophage efferocytosis via LRP1 in wound healing
Source: J Biomed Res. 2026 Mar 19;40(2):120–33. doi: 10.7555/JBR.38.20240375 (PMC13044401; doi:10.7555/JBR.38.20240375)
Supplement: Supplementary file 1 — Supplementary data to this article can be found online. [file jbr-40-2-120-S1.pdf]

Available online at www.jbr-pub.org.cn

Open Access at PubMed Central

Journal of Biomedical Research, 2026 40(2): 1–1

Supplementary Data

JBR

Remote neuromuscular electrical stimulation upregulates MDK to enhance macrophage efferocytosis *via* LRP1 in wound healing

Lijuan Zong[1,2,△], Chong Liu[2,△], Li Zhang[1,△], Xueyou Tao[3], Qingyan Tian[2], Xiaokai Zhou[4], Yu Wang[5], Na Shen[2], Jiaming Gong[2], Qingyuan Zhuang[2], Tong Wang[5], Wentao Liu[2,✉], Ying Shen[5,✉], Liang Hu[2,✉]

[1]Department of Anesthesiology, Children's Hospital of Nanjing Medical University, Nanjing, Jiangsu 210008, China;
[2]Department of Pharmacology, School of Basic Medical Sciences, Nanjing Medical University, Nanjing, Jiangsu 211166, China;
[3]Department of Anesthesiology, Yangzhou Maternal and Child Health Hospital Affiliated to Medical College of Yangzhou University, Yangzhou, Jiangsu 225001, China;
[4]Department of Anesthesia and Perioperative Medicine, the First Affiliated Hospital with Nanjing Medical University, Nanjing, Jiangsu 210029, China;
[5]Department of Rehabilitation, the First Affiliated Hospital of Nanjing Medical University, Nanjing, Jiangsu 210029, China.

Supplementary Videos 1–4 show that midkine (MDK) enhances the phagocytosis of apoptotic cells in macrophages. The online version contains supplementary videos available at http://www.jbr-pub.org.cn/article/doi/10.7555/JBR.38.20240375?pageType=en. Bone marrow-derived macrophages (BMDMs) were pretreated with lipopolysaccharide (LPS; 100 ng/mL) and interferon-γ (50 ng/mL) for 24 h. Apoptotic neutrophils (green) were added to BMDMs. BMDM phagocytosis in the control group (***Supplementary Video 1***). BMDM phagocytosis with LPS treated for 24 h (***Supplementary Video 2***). BMDM phagocytosis with LPS treated for 24 h and recombinant MDK (rMDK; 200 ng/mL) for 12 h (***Supplementary Video 3***). BMDM phagocytosis following 12-h rMDK-only (200 ng/mL) treatment (***Supplementary Video 4***).

△These authors contributed equally to this work.

✉Corresponding authors: Wentao Liu and Liang Hu, Department of Pharmacology, School of Basic Medical Sciences, Nanjing Medical University, 101 Longmian Avenue, Nanjing, Jiangsu 211166, China. E-mail: painresearch@njmu.edu.cn (Liu) and lianghu@njmu.edu.cn (Hu); Ying Shen, Department of Rehabilitation, the First Affiliated Hospital of Nanjing Medical University, 300 Guangzhou Road, Nanjing, Jiangsu 210029, China. E-mail: shenying_1981@hotmail.com.

Received: 16 November 2024; Revised: 23 May 2025; Accepted: 28 May 2025; Available online: 30 May 2025; Published date: 19 March 2026

CLC number: R454.1, Document code: A

The authors reported no conflict of interests.

This is an open access article under the Creative Commons Attribution (CC BY 4.0) license, which permits others to distribute, remix, adapt and build upon this work, for commercial use, provided the original work is properly cited.

© 2026 by Journal of Biomedical Research.

https://doi.org/10.7555/JBR.38.20240375
